# Controllable In-Situ Growth of Silver Nanoparticles on Filter Paper for Flexible and Highly Sensitive SERS Sensors for Malachite Green Residue Detection

**DOI:** 10.3390/nano10050826

**Published:** 2020-04-26

**Authors:** Lingzi Zhang, Jun Liu, Guowei Zhou, Zhiliang Zhang

**Affiliations:** 1 Key Laboratory of Fine Chemicals in Universities of Shandong, School of Chemistry and Pharmaceutical Engineering, Qilu University of Technology (Shandong Academy of Sciences), Jinan 250353, China; zlzzhanglingzi@163.com (L.Z.); chgwzhou@126.com (G.Z.); 2 State Key Laboratory of Biobased Material and Green Papermaking, Qilu University of Technology (Shandong Academy of Sciences), Jinan 250353, China; 3 School of Light Industry Science and Engineering, Qilu University of Technology (Shandong Academy of Sciences), Jinan 250353, China

**Keywords:** surface-enhanced Raman scattering, silver nanoparticles, polydopamine, filter paper, malachite green

## Abstract

In this work, a series of highly flexible and sensitive surface-enhanced Raman scattering (SERS) substrates were fabricated by the in-situ growth of silver nanoparticles (AgNPs) on polydopamine (PDA) templated filter papers (FPs), based on mussel-inspired surface chemistry. The obtained FP@PDA@AgNPs strips exhibited high sensitivity and reproducibility with Rhodamine 6G (R6G) probe molecules, with a calculated detection limit of approximately 10^−10^ M. More critically, these FP@PDA@AgNPs strips could be used as outstanding flexible SERS sensors to quickly collect and detect malachite green (MG) residues on fish scales, crab shells and shrimp skins by a swabbing extraction method. The detection limits for MG residues were calculated to be approximately as low as 0.04635 pg/cm^2^, 0.06952 pg/cm^2^ and 0.09270 pg/cm^2^, respectively. This facile and efficient strategy could to be utilized as a universal approach to fabricating a variety of flexible, cheap and portable SERS sensors for surface contamination analysis, and has great potential in the environmental scientific analysis and food safety monitoring fields.

## 1. Introduction

Malachite green (MG), as an important triphenylmethane molecule, has been widely used as a fungicidal and antiprotozoal agent in the aquaculture industry [1]. It could effectively prevent infections by protozoa, bacteria and oomycetes, and significantly improve fish survival rates. Simultaneously, the triphenylmethane structure in the MG molecule is very toxic and could induce a series of potential carcinogenic, mutagenic, teratogenic and other pathological effects [2,3]. Hence, MG has been classified as a banned drug in many places, including the USA, the European Union and other countries [4]. However, MG is still frequently and illegally abused to control the fungi in aquaculture because there are currently no cost-effective alternatives [5]. As the consumption of aquaculture products grows, there is increasing concern about the impact of MG residues on food safety and potential environmental hazards. The conventional methods for MG residue detection are chromatography [6,7], fluorescence spectrometry [8], mass spectrometry [9], etc., and these methods are seriously affected by several inherent defects, such as being time-consuming and highly priced, and requiring toxic organic solvents and complicated sample pretreatment process.

Since surface-enhanced Raman scattering (SERS)can combine ultra-sensitive and unlabeled detection, it has recently garnered considerable attention in the analytical chemistry, biomedical diagnostics, food safety, environmental monitoring and national security fields [10,11,12,13,14,15,16]. The effect of SERS is greatly affected by the localized surface plasmon resonance (LSPR). LSPR could be excited by resonance among the metal nanoparticles, nanoholes and nanowells. These plasmon resonances are accompanied by the enhancement of the electromagnetic field, which could induce Raman enhancement based on the electromagnetic mechanism (EM) [17,18]. Therefore, SERS performance is extremely contingent on the plasmonic properties of metal nanostructures and determined by their shape, size and aggregation states, as well as their surrounding environment. In general, the conventional rigid SERS substrates, such as glass, silicon and anodic aluminum oxide (AAO), have various metal nanostructures produced through a delicate nanolithography, and demonstrate high sensitivity and reproducibility [19,20,21,22]. Despite their high sensitivity and viability, however, these SERS substrates are generally rigid and fragile, which are not suitable attributes for extracting analytes from surfaces of various shapes, thus severely restricting the in-situ detection of analytes from a real-world surface.

In order to solve these problems, some researchers have paid much attention to impregnating noble metal nanoparticles among paper fibers in recent years, and fabricating various paper-based SERS substrates through inkjet printing, screening printing, filtration, dip coating and physical vapor deposition [23,24]. Paper-supported SERS substrates have several prominent characters compared to traditional SERS substrates. Firstly, the paper-supported SERS substrates are biodegradable since the paper substrates are primarily composed of cellulosic fibers, and would be regarded as a type of environment-friendly material to construct various functional sensors [25,26]. In addition, the paper-based SERS substrates are portable and cost-efficient, and could be easily cut into different shapes and sizes according to the demands of their practical applications [27]. More importantly, the paper-supported SERS substrates possess excellent flexibility, so they could readily swab an irregularly-shaped surface and collect samples from a real-world surface to the maximum extent [28]. In spite of tremendous advances in paper-based SERS substrate fabrication, at present, it still remains a huge challenge to exploit a simple and convenient tactic to fabricate robust paper-supported SERS substrates for the sensitive, rapid and convenient detection of MG residues from the surfaces of the real world.

As an important type of mussel-inspired molecule, dopamine (DA) has been widely explored for a versatile surface modification approach due to its unique self-polymerization [29,30]. Under alkaline conditions, DA can spontaneously self-polymerize into polydopamine (PDA) and be coated on almost all kinds of surface material [31,32]. Since the surface of the PDA molecules is rich in catechol and amine groups, it has an excellent complexing ability and could reduce the metal ions in-situ to the corresponding metallic nanoparticles [33,34,35].Since SERS performance is closely dependent on the nanostructure morphology, the size and distribution of metal nanoparticles on the surface of the PDA film could be regulated by changing the concentration of the metal precursor solution and the reaction time to achieve optimum SERS results [36,37,38,39]. Simultaneously, filter paper (FP) fibers possess a three-dimensional interconnected framework, and have large specific surfaces, excellent permeability and superior mechanical flexibility. These outstanding features are very beneficial for the manufacture of a flexible SERS substrate.

Based on these excellent characters of PDA molecules and FP fibers, FP@PDA@silver nanoparticles (AgNP s) strips were fabricated by using PDA molecules as templates in this research to achieve the in-situ reduction of AgNPs on the filter papers. The growth density of AgNPs on the surface of FP fibers was controlled by adjusting the reaction between the catechol groups of the PDA molecules and the silver ions in [Ag(NH_3_)_2_]^+^ solutions. The FP@PDA@AgNPs strips demonstrated high SERS sensitivity and uniformity toward Rhodamine 6G(R6G) probe molecules, with a calculated detection limit of approximately 10^−10^ M. More importantly, the as-prepared FP@PDA@AgNPs strips could be utilized as SERS sensors to directly analyze the MG residues on the fish scales, crab shells and shrimp skins by a simple swabbing method. The SERS analysis results showed that the detection limits for MG residues were almost as low as 0.04635 pg/cm^2^, 0.06952 pg/cm^2^ and 0.09270 pg/cm^2^, respectively, and the MG Raman peak intensity at 1616 cm^−1^ showed a great linear relationship with the corresponding logarithmic concentration. Due to excellent flexibility and significant electromagnetic coupling effect, these FP@PDA@AgNPs strips not only could significantly enhance the collection efficiency for MG residues, but also achieve superior SERS sensitivity and reliability. These SERS substrates could serve as sensitive sensors for the fast on-site detection of trace fungicides and demonstrate enormous potential in the aquaculture, environment and bioscience fields.

## 2. Materials and Methods

### 2.1. Chemicals and Materials

Dopamine hydrochloride, tris-based, were purchased from Sigma-Aldrich (St. Louis, MO, USA). Malachite green (MG, 95%) was produced by the Aladdin Industrial corporation (Shanghai, China). R6G was purchased from Beijing Chemical Co. (Beijing, China). Silver nitrate (AgNO_3_) was obtained from Jinan qiguang trading Co., Ltd. (Jinan, China). Ethanol and acetone were supplied by Tianjin Kemiou Chemical Reagent Co., Ltd. (Tianjin, China). Filter papers were provided by the Fushun civil filter paper factory (Fushun, China). In all experiments, ultrapure (18.2 MΩ) water produced by the Milli-Q system (Shanghai, China) was used.

### 2.2. Decoration of Filter Paper Strips with Dopamine

The filter paper was cut into small strips (1.0 × 0.5 cm^2^) and washed by ultrasonication in acetone, deionized (DI) water and ethanol. Under magnetic agitation, DA was slowly added to Tris-HCl buffer (10 mM, pH = 8.5) to prepare 2 mg/mL solutions [30], and then the cleaned FP strips were added and magnetically stirred at room temperature for 12 h. In the presence of oxygen molecules, PDA functional layers were spontaneously formed on the surface of FP fibers through DA self-polymerization. Since the catechol groups of dopamine were oxidized to produce a lot of melanin, the color of FP strips changed from white to dark gray accompanying the spontaneous oxidation of dopamine (Figure S1a). The obtained FP strips were subsequently washed three times with DI water and ethanol, and no excess polydopamine was left on the surface of the filter paper. The FP strips, after PDA deposition, were denoted as FP@PDA strips.

### 2.3. In-situ Growth of AgNPs on the FP@PDA Strip Surfaces

Firstly, NH_3_·H_2_O was added slowly and dropwise to a 2 mg/mL silver nitrate solution, under continual stirring, until the solution became clear from turbidity, to prepare the [Ag(NH_3_)_2_]^+^ solutions. Subsequently, the FP@PDA strips were totally immersed into the [Ag(NH_3_)_2_]^+^ solutions at room temperature, and the reaction was conducted with a 100–200 r/min stirring speed. By regulating the reaction time at 4, 8 and 12 h, AgNPs with different particle sizes and different coverage could be generated on the surface of FP@PDA strips. Finally, the strips were washed three times with DI water and ethanol to remove the excess AgNPs on the surface of FP @ PDA, and dried for 30 min in the oven at 60 °C. The FP@PDA strips, after AgNPs growth, were denoted as FP@PDA@AgNPs strips.

### 2.4. SERS Sensitivity and Reproducibility Tests

The SERS sensitivity and reproducibility of FP@PDA@AgNPs strips were tested using R6G as the probe molecule. Firstly, a standard R6G solution with 10^−3^ M concentration was accurately prepared in a volumetric flask and diluted with DI water into a series of solutions in the range of 10^−4^–10^−10^ M. Then, FP@PDA@AgNPs strips were immersed into 200 µL of R6G aqueous solution with different concentrations and dried for 30 min in an oven at 50 °C before SERS measurement. The Raman spectra of FP@PDA@AgNPs strips were collected on a Renishaw inVia9(Renishaw, London, UK) with a laser power of 1 mW.

### 2.5. SERS Detection of MG Residues with FP@PDA@AgNPs Strips

**Figure 1:**
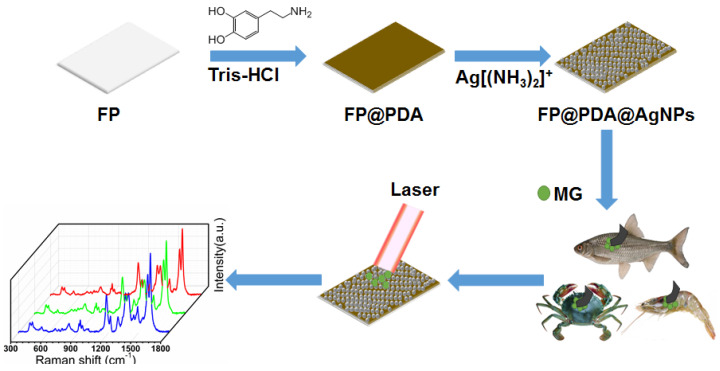
Schematic diagram of the preparation of flexible filter paper@polydopamine@silver nanoparticles (FP@PDA@AgNPs) surface-enhanced Raman spectroscopy (SERS) sensors for SERS detection of malachite green (MG) residues in aquatic products.

To simulate real-world SERS applications, the fish, crab and shrimps employed in the experiments were washed thoroughly with DI water and ethanol, and a certain volume of MG solutions with different concentrations (10^−3^–10^−10^ M) were evenly dropped onto the fish scales, crab shells and shrimp skins, respectively. Then, FP@PDA@AgNPs strips were attached onto the surfaces of fish scales, crab shells and shrimp skins respectively, and gentle swabbing was performed using tweezers to collect the MG residues from the surfaces. After the water evaporated, the FP@PDA@AgNPs strips were detected by Renishaw inVia9 with laser power of 10 mW to collect the Raman spectra. The fabrication procedure for FP@PDA@AgNPs strips and the detection of MG residues in aquatic products through swabbing extraction are illustrated in Figure 1.

### 2.6. Morphological and Chemical Characterization

The surface morphology of the above samples was characterized by a S-8220 scanning electron microscope (SEM, Hitachi, Tokyo, Japan) with an accelerating voltage of 5.0 kV. The chemical composition of nanostructures was determined by an energy-dispersive X-ray spectroscope (EDS, Bruker, Karlsruhe, Germany) attached to the SEM. The atomic force microscope (AFM) images were obtained in contact mode with a Multimode 8 atomic force microscope (Bruker, Karlsruhe, Germany). The crystalline structures of all samples were analyzed with a D8 Advance X-ray diffractometer (XRD, Bruker, Karlsruhe, Germany) using Cu Kα radiation (λ = 1.54 Å) at a voltage of 40 kV. X-ray photoelectron spectroscopy (XPS), with 200 W monochromatic Al Kα radiation, was performed, utilizing Thermo Scientific’sESCALabXi+ (Thermo Fisher, Waltham, MA, USA). Raman analyses were performed on a RenishawinVia9 (Renishaw, London, UK), using 532 nm laser irradiation, with a 50× objective focusing the laser beam onto a spot with a diameter of about 1 µm, and the collection time for each sample was about 5 s.

## 3. Results and Discussion

### 3.1. Morphology and Component Characterization of FP@PDA@AgNPs Strips

**Figure 2:**
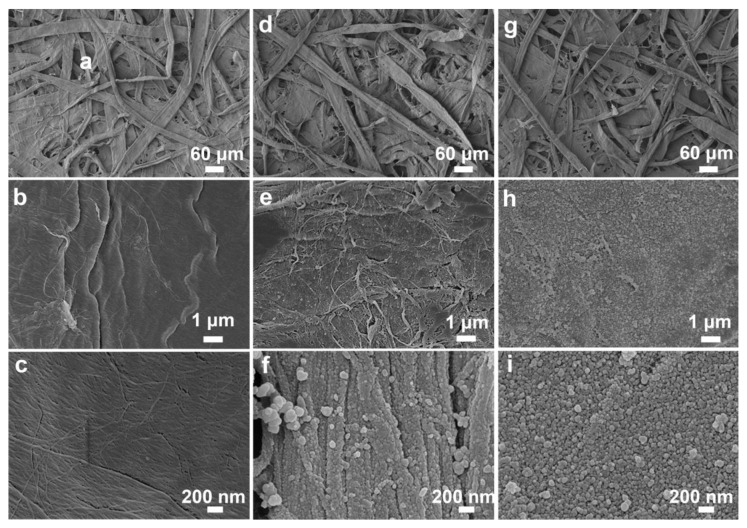
Scanning electron microscope(SEM) images of the FP strips (**a**–**c**), FP@PDA strips (**d**–**f**) and FP@PDA@AgNPs strips (**g**–**i**), respectively.

In order to confirm the successful self-polymerization of dopamine and in-situ growth of AgNPs on the surface of the FP strips, the surface morphology of the FP, FP@PDA and FP@PDA@AgNPs strips were examined using a scanning electron microscope (SEM) and atomic force microscope (AFM). From Figure 2a–c, it can be seen that the pristine FP strips revealed a three-dimensional interconnected framework and relatively smooth surface without any nanostructures. After wrapping a layer of PDA film, the FP@PDA strips showed a rough surface with some nanostructures emerging (Figure 2d–f). These nanostructures were probably attributable to the deposition of the PDA layer via self-polymerization and tight adsorption on the surface of FP@PDA strips. As they were further immersed into [Ag(NH_3_)_2_]^+^ solutions, abundant AgNPs were spontaneously formed via in-situ reduction and nearly covered the surface of the FP@PDA@AgNPs strips (Figure 2g–i).According to the statistical results for the SEM image with larger magnification (Figure S1b), it appears that the AgNPs had a size of about 50 ± 10 nm, with spherical morphology, and the AgNPs were uniformly dispersed on the FP strips and could produce a strong LSPR effect.

**Figure 3:**
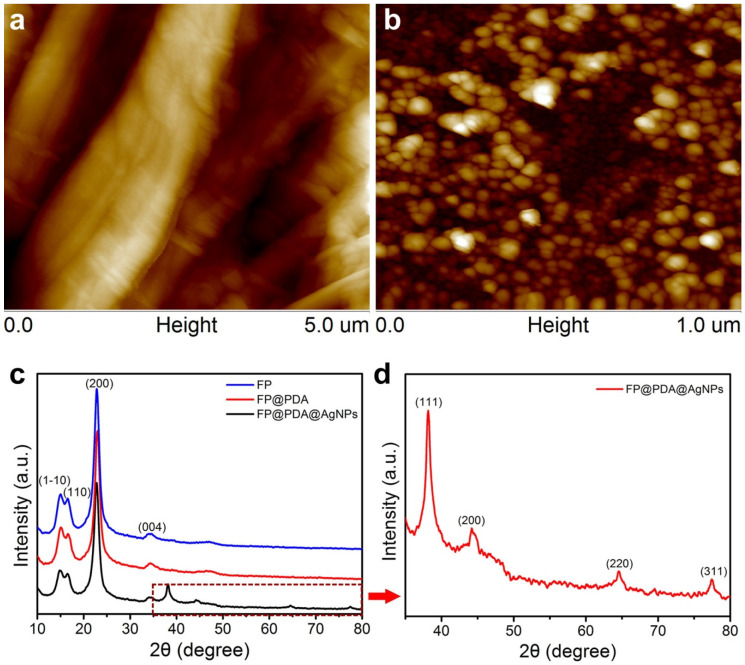
Atomic force microscope (AFM) images of the (**a**) FP strips, and (**b**) FP@PDA@AgNPs strips, and (**c**) X-ray diffraction (XRD) spectra of the pristine FP, FP@PDA, and FP@PDA@AgNPs strips. (**d**) High resolution XRD spectrum of the FP@PDA@AgNPs strips.

As shown in Figure 3a–b, by comparing the AFM image of the pristine FP strips and those of the FP@PDA@AgNPs strips, it was also found that AgNPs spontaneously formed by in-situ reduction and almost covered the surface of the FP@PDA@AgNPs strips, which was consistent with the SEM analysis results (Figure 2a–f). The crystal structures of the pristine FP, FP@PDA and FP@PDA@AgNPs strips were monitored by XRD and are shown in Figure 3c. The XRD pattern of the pristine FP strips presented four obvious diffraction peaks at 15.0°, 16.6°, 22.7° and 34.3°, which originated from the (1–10), (110), (200) and (004) planes of cellulose fibers, respectively [37]. Compared with the pristine FP strips, no additional diffraction peaks emerged in the XRD spectrum of the FP@PDA strips, and this was primarily owing to the amorphous structure of PDA during the self-polymerization process. By contrast, as seen from Figure 3d, four more characteristic peaks appeared at 38.1°, 44.7°, 64.9° and 77.4° in the XRD pattern of the FP@PDA@AgNPs strips, corresponding to (111), (211) and (311) planes of face centered cubic (fcc) of AgNPs (JCPDS No.83-0718), respectively [40]. The elemental changes in the pristine FP, FP@PDA and FP@PDA@AgNPs strips were also characterized by an energy spectrometer and are shown in Figure S2a–c. It could be seen that after FP@PDA strips were put into [Ag(NH_3_)_2_]^+^ solution, the silver peak appeared and exhibited a significant peak intensity due to a large amount of AgNPs formation on the surface of the FP@PDA@AgNPs strips. These EDS analyses were consistent with the XRD results and proved that a good number of AgNPs were successfully formed on the surface of the FP@PDA@AgNPs strips.

**Figure 4:**
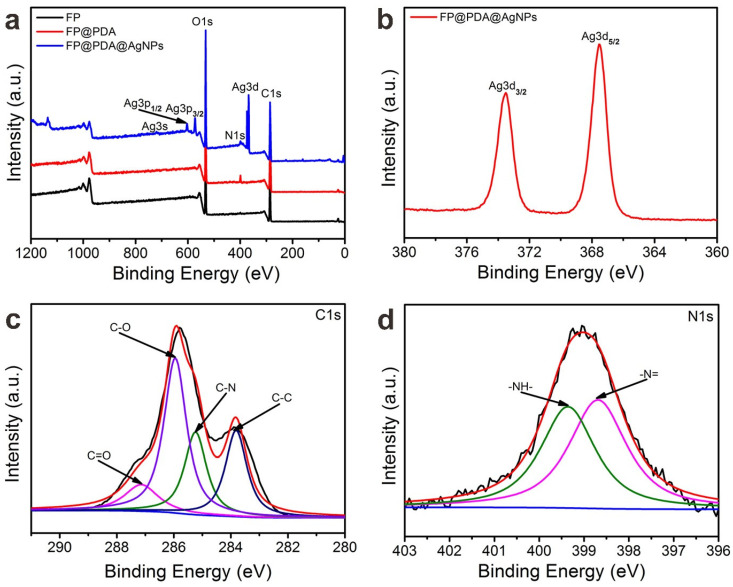
(**a**) X-ray photoelectron spectra (XPS) of the pristine FP, FP@PDA, and FP@PDA@AgNPs strips. (**b**) Ag3d spectra of FP@PDA@AgNPs. High-resolution (**c**) C1s spectrum and (**d**) N1s spectrum of the FP@PDA strips.

To further confirm the chemical composition, the pristine FP, FP@PDA and FP@PDA@AgNPs strips were characterized using XPS, and Figure 4 shows the respective XPS spectra. As shown in Figure 4a, all materials showed XPS signals with binding energies (BE) of 285.95 and 532.2 eV, corresponding to the C1s, N1s and O1s, respectively. Compared with the XPS results of the original FP and FP@PDA strips, the XPS spectra of the FP@PDA@AgNPs strips showed obvious emission peaks characteristic of Ag3d (367.55 and 373.50 eV), Ag3p (573 and 603 eV) and Ag3s (721 eV). As shown in Figure 4b, the detailed map of the Ag3d spectrum showed two main peaks at 367.55 eV and 373.50 eV, which could be identified as Ag3d_5/2_ and Ag3d_3/2_, respectively [40]. These results indicated that AgNPs had successfully covered the surface of FP@PDA strips, and that the coated AgNPs were present in a metallic state. In addition, Figure 4c,d shows the high-resolution C1s and N1s spectra of the FP@PDA strips. As shown in Figure 4c, the C1s high-resolution XPS spectra were fitted and divided into four peaks (283.80, 285.25, 285.95 and 287.15 eV), attributable to the binding energy peaks of C–C bonds, C–N groups, C–O groups and carbonyl groups (C=O), respectively. Similarly, the high resolution XPS image of N1s was also fitted and split into two peaks (398.70 and 399.35 eV) representing –N= and –NH– group, respectively. The above analysis fully proved that the PDA was successfully wrapped on the filter paper by DA self-polymerization.

### 3.2. SERS Performance of the FP@PDA@AgNPs Strips with R6G as a Probe Molecule

**Figure 5:**
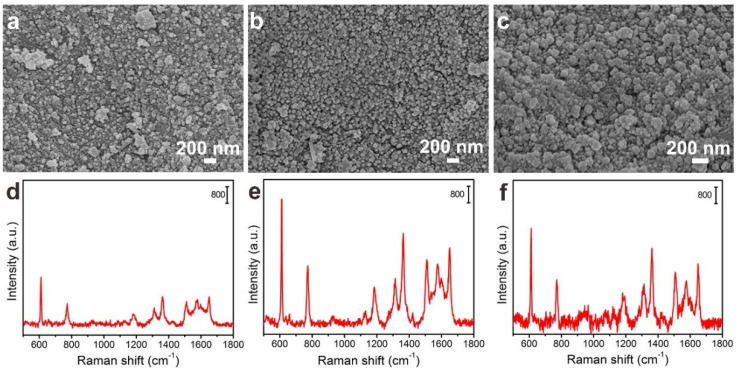
Images of AgNPs on FP@PDA with different reaction times: (**a**) 4 h, (**b**) 8 h and (**c**) 12 h. (**d**–**f**) SERS spectra of Rhodamine 6G (R6G) (10^−6^ M) collected for the corresponding 4h, 8h and 12h FP@PDA@AgNPs strips.

In order to obtain the best SERS substrates, the effect of the reaction time of FP@PDA in [Ag(NH_3_)_2_]^+^ solutions on their surface morphology was studied, and the intensity of SERS signal was tested with R6G at a concentration of 10^−6^ M as a probe molecule. As shown in Figure 5, the size, shape and density of AgNPs varied with reaction time. In the case of a 4 h reaction time (Figure 5a), only a few AgNPs with a size below 30 nm were distributed on the surface of the FP@PDA@AgNPs strips. Accordingly, such a substrate exhibited a low SERS effect (Figure 5d). After the reaction lasting for 8 h, it was found that as the in-situ reduction time of catechol groups increased, more and more [Ag(NH_3_)_2_]^+^ ions were converted into AgNPs, and the size of AgNPs gradually reached 50–60 nm (Figure 5b). Consequently, many nanogaps between adjacent AgNPs were formed and could be served as SERS “hot spots” to induce the strong LSPR effect and produce significant Raman signals (Figure 5e). However, as the reaction time further increased to 12 h, the nanoparticle size became bigger and uneven (Figure 5c) and the intensity of the Raman signal demonstrated an obvious decline trend (Figure 5f). As a result, the FP@PDA@AgNPs strips fabricated for 8 h would be utilized as flexible SERS substrates to detect R6G and MG molecules in the following SERS experiments.

**Figure 6:**
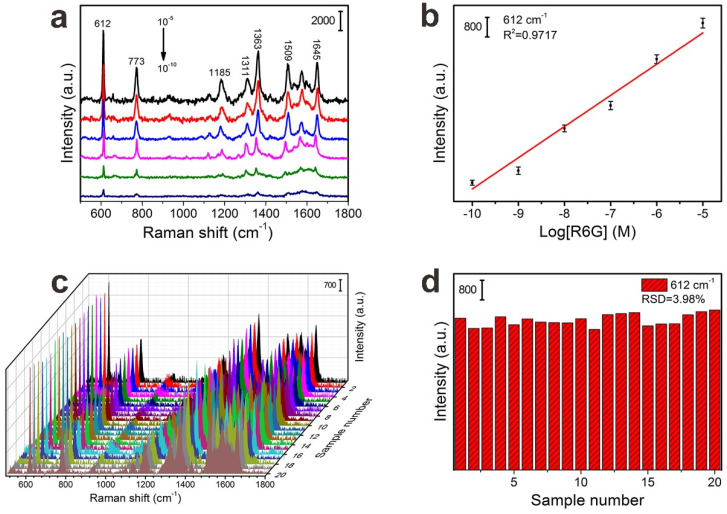
(**a**) Spectra of R6G with 10^−5^ M, 10^−^^6^ M, 10^−^^7^ M, 10^−^^8^ M, 10^−^^9^ M and 10^−10^ M concentrations, collected from the respective FP@PDA@AgNPs strips. (**b**) The linear relationship between the Raman intensity at 612 cm^−1^ and the logarithm values of R6G concentration from 10^−5^ to 10^−10^ M. (**c**) SERS spectra of 10^−6^ M R6G collected from 20 randomly selected spots on the FP@PDA@AgNPs strips. (**d**) Intensity distribution of characteristic Raman peaks at 612 cm^−1^.

The sensitivity and reproducibility of the SERS substrate were essential to achieve trace and reliable detection for target molecules. Figure 6 shows the SERS spectra of R6G molecules in the range of 10^−5^ to 10^−10^ M on FP@PDA@AgNPs strips, and clearly observed are the characteristic Raman peaks at 612, 773, 1185, 1311, 1363, 1509 and 1645 cm^−1^.Among them, the Raman peaks located at 612 and 773 cm^−1^ were attributable to the C–C–C ring in-plane bending mode of vibration and the out-of-plane bending motion of the hydrogen atoms, respectively, whereas the peaks at 1363, 1509 and 1645 cm^−1^ were associated with the aromatic C–C stretching vibrations of the R6G molecules, which was consistent with previous reports regarding R6G [41,42]. As shown in Figure 6a, as the concentration of R6G decreased from 10^−5^ M to 10^−10^ M, the SERS Raman peaks intensity dropped dramatically. Even though the concentration was decreased to 10^−10^ M, the characteristic peaks of R6G at 612 and 773 cm^−1^ were clearly observed, which indicated that the FP@PDA@AgNPs strips, as SERS sensors, had enough sensitivity to detect trace probe molecules. As seen from Figure S2d, the limit of detection concentration of the FP@PDA@AgNPs strips for R6G could reach 10^−10^ M. In addition, Figure 6b displayed that the strength of the Raman peak at 612 cm^−1^ had a good linear relationship with the logarithm of the R6G concentration, and the correlation coefficient (*R*^2^) was 0.9717, which would be very advantageous for being able to determine the concentration of target molecules with these highly sensitive FP@PDA@AgNPs SERS strips.

Apart from high sensitivity, the uniformity of the SERS substrate was another indispensable parameter for practical application. To check the uniformity, the SERS signals of R6G with a concentration of 10^−6^ M were collected from 20 randomly selected spots from the FP@PDA@AgNPs strips. As can be seen from Figure 6c, all selected spots revealed relatively consistent Raman signals at 612, 773, 1185, 1311, 1363, 1509 and 1645 cm^−1^, and the relative standard deviations (RSD) of the Raman vibration at 612 cm^−1^ were approximately 3.98% (Figure 6d). The above results proved that the FP@PDA@AgNPs strips could be utilized as a highly uniform SERS substrate.

To estimate the SERS performance of the FP@PDA@AgNPs strips, the enhancement factor (*EF*) was calculated as follows:

(1) $$EF=\frac{I_{\mathrm{SERS}}N_{\mathrm{RS}}}{I_{\mathrm{RS}}N_{\mathrm{SERS}}}$$

where *I*_SERS_ and I_RS_ were the peak intensities of the R6G molecule SERS spectrum and R6G molecule normal Raman spectrum at 612 cm^−1^, respectively. *N*_SERS_ and *N*_RS_ were equal to the numbers of R6G molecules on the respective substrates within the laser spots [43]. According to Figure S3a and the calculation method in the supporting information, the EF of the FP@PDA@AgNPs strips was as high as1.78 × 10^7^ for the R6G probe molecules, indicating a Raman enhancement effect superior to those in previous reports [44,45].

### 3.3. Swab Detection of MG Residues with FP@PDA@AgNPs Strips

**Figure 7:**
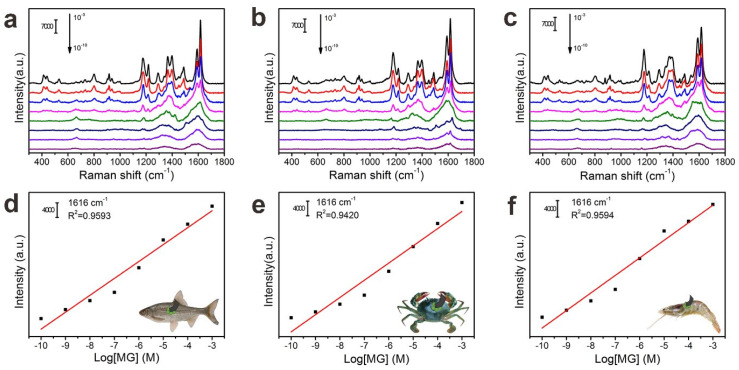
Spectra of MG with 10^−^^3^ M, 10^−^^4^ M, 10^−5^ M, 10^−^^6^ M, 10^−^^7^ M, 10^−^^8^ M, 10^−^^9^ M and 10^−10^ M concentrations collected from (**a**) fish scales, (**b**) crab shells and (**c**) shrimp skins using the FP@PDA@AgNPs strips by swabbing extraction. The linear plots between the SERS peak intensity at 1616 cm^−1^ and the logarithm of MG concentration from (**d**) fish scales, (**e**) crab shells and (**f**) shrimp skin surface, using the FP@PDA@AgNPs strips by swabbing extraction.

Due to their good flexibility, FP@PDA@AgNPs strips were expected to be used as SERS sensors for the fast on-site and ultra-sensitive detection of contaminants on a wide range of objects. To demonstrate the possibility of using FP@PDA@AgNPs strips for detecting MG residues in the aquaculture industry, the MG molecules were dissolved in DI water and diluted to solutions with concentrations of 10^−3^–10^−10^ M. The diluted MG solutions were sequentially dropped on the surfaces of fish scales, crab shells and shrimp skins. The MG residues were collected by surface swabbing using the prepared FP@PDA@AgNPs strips. Figure 7a–c illustrates that all of the dominating characteristic bands of MG molecules clearly appeared in the region from 300 to 1800 cm^−1^. The Raman peaks located at 414, 800, 916, 1175 and 1616 cm^−1^ were attributed to the in-plane vibration of phenyl–C–phenyl, ring C–H out-of-plane bending, C–H out-of-plane bending and ring skeletal vibration, in-plane vibrations of ring C–H, and ring C–C stretching vibration, which was very consistent with a previous SERS report regarding MG [46].

As the concentration decreased from 10^−3^ to 10^−10^ M, the peak intensity of MG dropped sharply. Even if the concentration was reduced to 10^−10^ M, the characteristic peaks of MG at 1175 and 1616 cm^−1^ could still be observed, which indicated that the FP@PDA@AgNPs strips processed high SERS sensitivity for MG residue detection. According to the MG concentration and the volume sprayed onto the fish scales, crab shells and shrimp skins, the detection limit for MG residues was calculated to be approximately as low as 0.04635 pg/cm^2^, 0.06925 pg/cm^2^ and 0.09270 pg/cm^2^, by the swabbing extraction method (Figure 7d–f). As shown in Figure S3b–d, according to the previous calculation method, the EFs of the FP@PDA@AgNPs strips for the MG on the fish scales, crab shells and shrimps’ skins were calculated to be 4.06 × 10^6^, 2.85 × 10^6^ and 1.99 × 10^6^, respectively, which were higher than those from related methods [47,48]. Furthermore, the peak intensity of MG at 1616 cm^−1^ showed a good linear relationship with the corresponding logarithmic concentration, and the *R*^2^ values for MG on fish scales, crab shells and shrimp skins were 0.9593, 0.9420 and 0.9594, respectively. These analyses fully demonstrated that the FP@PDA@AgNPs strips could be used as flexible SERS sensors to efficiently and sensitively detect MG residues on the surfaces of aquatic products.

**Figure 8:**
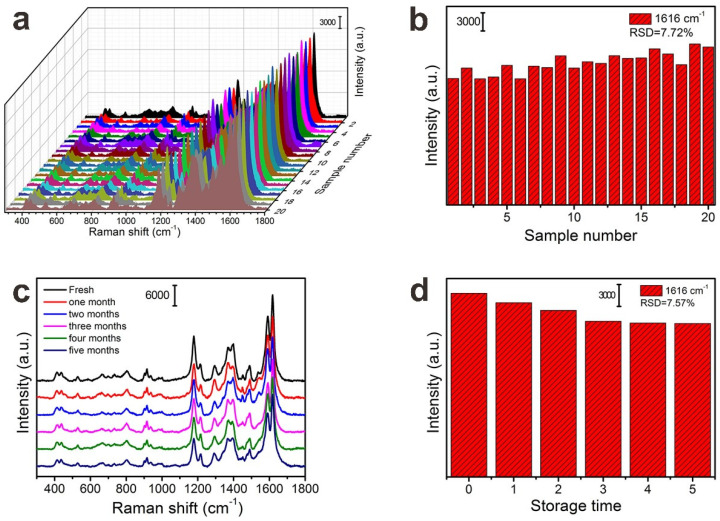
(**a**) SERS spectra of 10^−5^ M MG collected from 20 randomly selected spots on the FP@PDA@AgNPs strips. (**b**) Intensity distribution of characteristic Raman peaks at 1616 cm^−1^. (**c**) SERS spectra of 10^−5^ M MG were collected on the FP@PDA@AgNPs strips with different storage times of 0–5 months. (**d**) SERS intensity of MG at 1616 cm^−1^ after different storage times.

In addition to the sensitivity, the uniformity of the FP@PDA@AgNPs strips was also tested, and the Raman signals of MG (10^−5^ M) were detected from 20 points randomly selected on the FP@PDA@AgNPs strips. As can be observed in Figure 8a, all selected points at 437, 800, 916, 1175 and 1616 cm^−1^ showed relatively consistent Raman signals, and the RSD of Raman vibration at 1616 cm^−1^ was approximately 7.72% (Figure 8b). Furthermore, the stability of the SERS substrate was also a very important parameter for obtaining reliable Raman detection. As shown in Figure 8c–d, the Raman signal did not decrease significantly compared to the SERS spectra from fresh samples, even though the FP@PDA@AgNPs strips had been stored for five months. The RSD values of the Raman peaks at 1616 cm^−1^ were approximately 7.75%, and these results indicated that the Raman signals on the FP@PDA@AgNPs strips demonstrated good SERS stability. The above results proved that the flexible FP@PDA@AgNPs strips processed superior sensitivity, uniformity and storage stability. In addition to detecting MG residues in aquatic products, these flexible SERS substrates would be expected to be usable as highly sensitive SERS sensors to monitor various pesticides, explosives and carcinogens in the food safety and science fields.

## 4. Conclusions

In a word, PDA was used as a versatile molecular platform for the in-situ reduction of AgNPs on the filter papers, based on mussel-inspired surface chemistry, and a series of flexible FP@PDA@AgNPs strips were fabricated. Due to high sensitivity and excellent reproducibility, the FP@PDA@AgNPs strips could produce stable and reliable Raman signals from R6G probe molecules, even if the concentration of R6G was as low as 10^−10^ M. More importantly, the FP@PDA@AgNPs strips could be used as flexible SERS sensors to rapidly collect and detect MG residues on the surfaces of fish scales, crab shells and shrimp skins via a simple swabbing method. The detection limits for MG residues were calculated to be nearly as low as 0.04635 pg/cm^2^, 0.06952 pg/cm^2^ and 0.09270 pg/cm^2^, respectively, and the peak intensity at 612 cm^−1^ for the MG residues illustrated a good linear relationship with the corresponding logarithmic concentration. These designed FP@PDA@AgNPs strips would show great application potential for the fast on-site detection of trace target molecules in the aquaculture, environment and bioscience fields.

## Supplementary Materials

The following are available online at https://www.mdpi.com/2079-4991/10/5/826/s1, Figure S1: (**a**) Optical images of the original filter paper strip, FP@PDA strip and FP@PDA@AgNPs strip. (**b**) SEM images of the FP@PDA@AgNPs. Figure S2: The responding EDS of (**a**) FP (**b**) FP@PDA and (**c**) FP@PDA@AgNPs respectively. (**d**) The magnified SERS spectrum of R6G molecules with concentration at 10^−10^ M. Figure S3: (**a**) SERS spectra (black) of 200 µL 10^−10^ M R6G on 50 mm^2^ FP@PDA@AgNPs and Raman spectrum (red) of 10^−3^ M R6G on 50 mm^2^ silicon wafer. SERS spectra (black) of 50, 75, 100 µL 10^−9^ M MG on 50 mm^2^ FP@PDA@AgNPs swabs collected from (**b**) fish scales, (**c**) crab shells and (**d**) shrimp skins surface and Raman spectrum (red) of 50 µL 10^−3^ M MG on 50 mm^2^ silicon wafer.

## References

[1] Ouyang L., Yao L., Zhou T., Zhu L. (2018). Accurate SERS detection of malachite green in aquatic products on basis of graphene wrapped flexible sensor. Anal. Chim. Acta.

[2] Zhao X., Yu J., Zhang C., Chen C., Xu S., Li C., Li Z., Zhang S., Liu A., Man B. (2018). Flexible and stretchable SERS substrate based on a pyramidal PMMA structure hybridized with graphene oxide assivated AgNPs. Appl. Surf. Sci..

[3] Kumar P., Khosla R., Soni M., Deva D., Sharma S.K. (2017). A highly sensitive, flexible SERS sensor for malachite green detection based on Ag decorated microstructured PDMS substrate fabricated from Taro leaf as template. Sens. Actuators B Chem..

[4] Zhang C., Li C., Yu J., Jiang S., Xu S., Yang C., Liu Y.J., Gao X., Liu A., Man B. (2018). SERS activated platform with three-dimensional hot spots and tunable nanometer gap. Sens. Actuators B Chem..

[5] Yang N., You T.-T., Gao Y.-K., Zhang C.-M., Yin P.-G. (2018). Fabrication of a Flexible Gold Nanorod Polymer Metafilm via a Phase Transfer Method as a SERS Substrate for Detecting Food Contaminants. J. Agric. Food Chem..

[6] Chen G., Miao S. (2010). HPLC Determination and MS Confirmation of Malachite Green, Gentian Violet, and Their Leuco Metabolite Residues in Channel Catfish Muscle. J. Agric. Food Chem..

[7] Maxwell E.J., Tong W.G. (2016). Sensitive detection of malachite green and crystal violet by nonlinear laser wave mixing and capillary electrophoresis. J. Chromatogr. B.

[8] Wu L., Lin Z.-Z., Zhong H.-P., Chen X.-M., Huang Z.-Y. (2017). Rapid determination of malachite green in water and fish using a fluorescent probe based on CdTe quantum dots coated with molecularly imprinted polymer. Sens. Actuators B Chem..

[9] Halme K., Lindfors E., Peltonen K. (2007). A confirmatory analysis of malachite green residues in rainbow trout with liquid chromatography–electrospray tandem mass spectrometry. J. Chromatogr. B.

[10] Shi Y.-E., Li L., Yang M., Jiang X., Zhao Q., Zhan J. (2014). A disordered silver nanowires membrane for extraction and surface-enhanced Raman spectroscopy detection. Analyst.

[11] Huang Z., Zhang R., Chen H., Weng W., Lin Q., Deng D., Li Z., Kong J. (2019). Sensitive polydopamine bi-functionalized SERS immunoassay for microalbuminuria detection. Biosens. Bioelectron..

[12] Luo W., Chen M., Hao N., Huang X., Zhao X., Zhu Y., Yang H., Chen X. (2019). In situ synthesis of gold nanoparticles on pseudo-paper films as flexible SERS substrate for sensitive detection of surface organic residues. Talanta.

[13] Fan M., Zhang Z., Hu J., Cheng F., Wang C., Tang C., Lin J., Brolo A.G., Zhan H. (2014). Ag decorated sandpaper as flexible SERS substrate for direct swabbing sampling. Mater. Lett..

[14] Zhang Z., Si T., Liu J., Zhou G. (2019). In-Situ Grown Silver Nanoparticles on Nonwoven Fabrics Based on Mussel-Inspired Polydopamine for Highly Sensitive SERS Carbaryl Pesticides Detection. Nanomaterials.

[15] Liu J., Si T., Zhang Z. (2019). Mussel-inspired immobilization of silver nanoparticles toward sponge for rapid swabbing extraction and SERS detection of trace inorganic explosives. Talanta.

[16] Liu J., Si T., Zhang L., Zhang Z. (2019). Mussel-Inspired Fabrication of SERS Swabs for Highly Sensitive and Conformal Rapid Detection of Thiram Bactericides. Nanomaterials.

[17] Zhang M., Chen T., Liu Y., Zhang J., Sun H., Yang J., Zhu J., Liu J., Wu Y. (2018). Plasmonic 3D Semiconductor–Metal Nanopore Arrays for Reliable Surface-Enhanced Raman Scattering Detection and In-Site Catalytic Reaction Monitoring. ACS Sens..

[18] Tong Q., Wang W., Fan Y., Dong L. (2018). Recent progressive preparations and applications of silver-based SERS substrates. TrAC Trends Anal. Chem..

[19] Tang J., Chen W., Ju H. (2019). Rapid detection of pesticide residues using a silver nanoparticles coated glass bead as nonplanar substrate for SERS sensing. Sens. Actuators B Chem..

[20] Wang P., Zhou Y., Wen Y., Wang F., Yang H. (2015). In situ polydopamine-assisted deposition of silver nanoparticles on a two dimensional support as an inexpensive and highly efficient SERS substrate. RSC Adv..

[21] Kim A.N., Lim H., Lee H.N., Park Y.M., Yoo B., Kim H.-J. (2018). Large-area and cost-effective fabrication of Ag-coated polymeric nanopillar array for surface-enhanced Raman spectroscopy. Appl. Surf. Sci..

[22] Zhu C., Meng G., Zheng P., Huang Q., Li Z., Hu X., Wang X., Huang Z., Li F., Wu N. (2016). A Hierarchically Ordered Array of Silver-Nanorod Bundles for Surface-Enhanced Raman Scattering Detection of Phenolic Pollutants. Adv. Mater..

[23] Ma Y., Wang Y., Luo Y., Duan H., Li D., Xu H., Fodjo E.K. (2018). Rapid and sensitive on-site detection of pesticide residues in fruits and vegetables using screen-printed paper-based SERS swabs. Anal. Methods.

[24] Lee M., Oh K., Choi H.-K., Lee S.G., Youn H.J., Lee H.L., Jeong D.H. (2018). Subnanomolar Sensitivity of Filter Paper-Based SERS Sensor for Pesticide Detection by Hydrophobicity Change of Paper Surface. ACS Sens..

[25] Kwon G., Kim J., Kim D., Ko Y., Yamauchi Y., You J. (2019). Nanoporous cellulose paper-based SERS platform for multiplex detection of hazardous pesticides. Cellulose.

[26] Polavarapu L., Porta A.L., Novikov S.M., Coronado-Puchau M., Liz-Marzán L.M. (2014). Pen-on-Paper Approach toward the Design of Universal Surface Enhanced Raman Scattering Substrates. Small.

[27] Lee C.H., Hankus M.E., Tian L., Pellegrino P.M., Singamaneni S. (2011). Highly Sensitive Surface Enhanced Raman Scattering Substrates Based on Filter Paper Loaded with Plasmonic Nanostructures. Anal. Chem..

[28] Prikhozhdenko E.S., Bratashov D.N., Gorin D.A., Yashchenok A.M. (2018). Flexible surface-enhanced Raman scattering-active substrates based on nanofibrous membranes. Nano Res..

[29] Ye Q., Zhou F., Liu W. (2011). Bioinspired catecholic chemistry for surface modification. Chem. Soc. Rev..

[30] Lee H., Dellatore S.M., Miller W.M., Messersmith P.B. (2007). Mussel-Inspired Surface Chemistry for Multifunctional Coatings. Science.

[31] Lee B.P., Messersmith P.B., Israelachvili J.N., Waite J.H. (2011). Mussel-Inspired Adhesives and Coatings. Ann. Rev. Mater. Res..

[32] Yan J., Yang L., Lin M.-F., Ma J., Lu X., Lee P.S. (2013). Polydopamine Spheres as Active Templates for Convenient Synthesis of Various Nanostructures. Small.

[33] Liu Y., Ai K., Lu L. (2014). Polydopamine and Its Derivative Materials: Synthesis and Promising Applications in Energy, Environmental, and Biomedical Fields. Chem. Rev..

[34] Saiz-Poseu J., Mancebo-Aracil J., Nador F., Busqué F., Ruiz-Molina D. (2019). The Chemistry behind Catechol-Based Adhesion. Angew. Chem. Int. Ed..

[35] Wang W., Jiang Y., Wen S., Liu L., Zhang L. (2012). Preparation and characterization of polystyrene/Ag core–shell microspheres—A bio-inspired poly(dopamine) approach. J. Colloid Interface Sci..

[36] Shang B., Wang Y., Yang P., Peng B., Deng Z. (2018). Synthesis of superhydrophobic polydopamine-Ag microbowl/nanoparticle array substrates for highly sensitive, durable and reproducible surface-enhanced Raman scattering detection. Sens. Actuators B Chem..

[37] Cheng D., Bai X., He M., Wu J., Yang H., Ran J., Cai G., Wang X. (2019). Polydopamine-assisted immobilization of Ag@AuNPs on cotton fabrics for sensitive and responsive SERS detection. Cellulose.

[38] Wang P., Zhou Y., Zhou Y., Wen Y., Wang F., Yang H. (2017). In-situ growth of raspberry-like silver composites for Raman detection of acrylamide. Sens. Actuators B Chem..

[39] Xu F., Xie S., Cao R., Feng Y.N., Ren C., Wang L. (2017). Prepare poly-dopamine coated graphene@silver nanohybrid for improved surface enhanced Raman scattering detection of dyes. Sens. Actuators B Chem..

[40] Han K., Liu Y., Huang H., Gong Q., Zhang Z., Zhou G. (2019). Tremella-like NiO microspheres embedded with fish-scale-like polypyrrole for high-performance asymmetric supercapacitor. RSC Adv..

[41] Zhang K., Zhao J., Xu H., Li Y., Ji J., Liu B. (2015). Multifunctional Paper Strip Based on Self-Assembled Interfacial Plasmonic Nanoparticle Arrays for Sensitive SERS Detection. ACS Appl. Mater. Interfaces.

[42] Li Y., Zhang K., Zhao J., Ji J., Ji C., Liu B. (2016). A three-dimensional silver nanoparticles decorated plasmonic paper strip for SERS detection of low-abundance molecules. Talanta.

[43] Huang Z., Meng G., Huang Q., Yang Y., Zhu C., Tang C. (2010). Improved SERS Performance from Au Nanopillar Arrays by Abridging the Pillar Tip Spacing by Ag Sputtering. Adv. Mater..

[44] Kalachyova Y., Erzina M., Postnikov P., Svorcik V., Lyutakov O. (2018). Flexible SERS substrate for portable Raman analysis of biosamples. Appl. Surf. Sci..

[45] Yang L., Hu J., He L., Tang J., Zhou Y., Li J., Ding K. (2017). One-pot synthesis of multifunctional magnetic N-doped graphene composite for SERS detection, adsorption separation and photocatalytic degradation of Rhodamine 6G. Chem. Eng. J..

[46] Chen X., Nguyen T.H.D., Gu L., Lin M. (2017). Use of Standing Gold Nanorods for Detection of Malachite Green and Crystal Violet in Fish by SERS. J. Food Sci..

[47] Zhao J., Lin J., Li X., Zhao G., Zhang W. (2015). Silver nanoparticles deposited inverse opal film as a highly active and uniform SERS substrate. Appl. Surf. Sci..

[48] Ogundare S.A., van Zyl W.E. (2019). Amplification of SERS “hot spots” by silica clustering in a silver-nanoparticle/nanocrystalline-cellulose sensor applied in malachite green detection. Colloid Surf. A-Physicochem. Eng. Asp..

