# Supplementary material for: Controllable In-Situ Growth of Silver Nanoparticles on Filter Paper for Flexible and Highly Sensitive SERS Sensors for Malachite Green Residue Detection
Source: Nanomaterials (Basel). 2020 Apr 26;10(5):826. doi: 10.3390/nano10050826 (PMC7712161; doi:10.3390/nano10050826)
Supplement: Supplementary file 1 [file nanomaterials-10-00826-s001.pdf]

Supporting information

Controllable In-Situ Growth of Silver Nanoparticles on Filter Paper for Flexible and Highly Sensitive SERS Sensors for Malachite Green Residue Detection

Lingzi Zhang[1], Jun Liu [2,3,*], Guowei Zhou[1] and Zhiliang Zhang[1,2,*]

[1] Key Laboratory of Fine Chemicals in Universities of Shandong, School of Chemistry and Pharmaceutical Engineering, Qilu University of Technology (Shandong Academy of Sciences), Jinan 250353, China; zlzzhanglingzi@163.com (L.Z.); chgwzhou@126.com (G.Z.)

[2] State Key Laboratory of Biobased Material and Green Papermaking, Qilu University of Technology (Shandong Academy of Sciences), Jinan 250353, China

[3] School of Light Industry Science and Engineering, Qilu University of Technology (Shandong Academy of Sciences), Jinan 250353, China

*Correspondence: liujun6621@126.com (J.L.); zhzhl@iccas.ac.cn (Z.Z.); Tel.: +86-053189631632

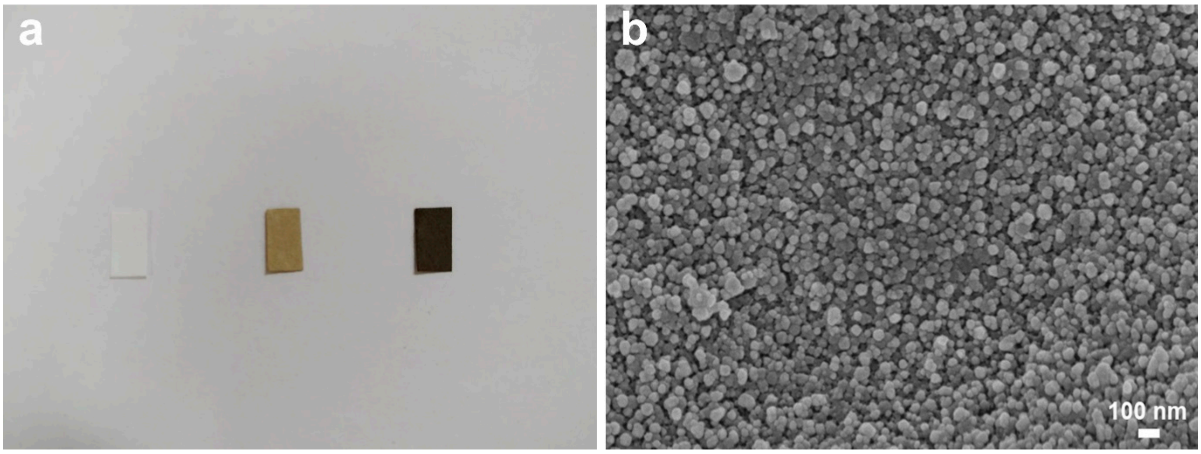

Figure S1. (**a**) Optical images of the original filter paper strip, FP@PDA strip and FP@PDA@AgNPs strip. (**b**) SEM images of the FP@PDA@AgNPs.

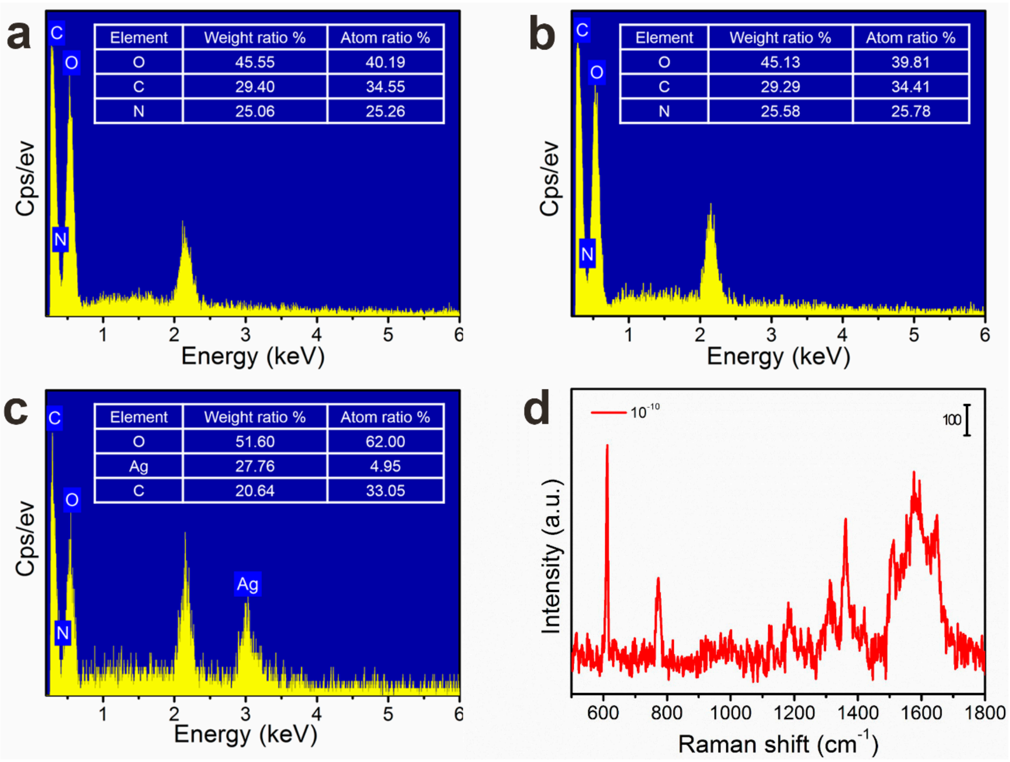

Figure S2. The corresponding EDS for (**a**) FP, (**b**) FP@PDA and (**c**) FP@PDA@AgNPs, respectively. (**d**) The magnified SERS spectrum for R6G molecules with a concentration of 10^{-10} M.

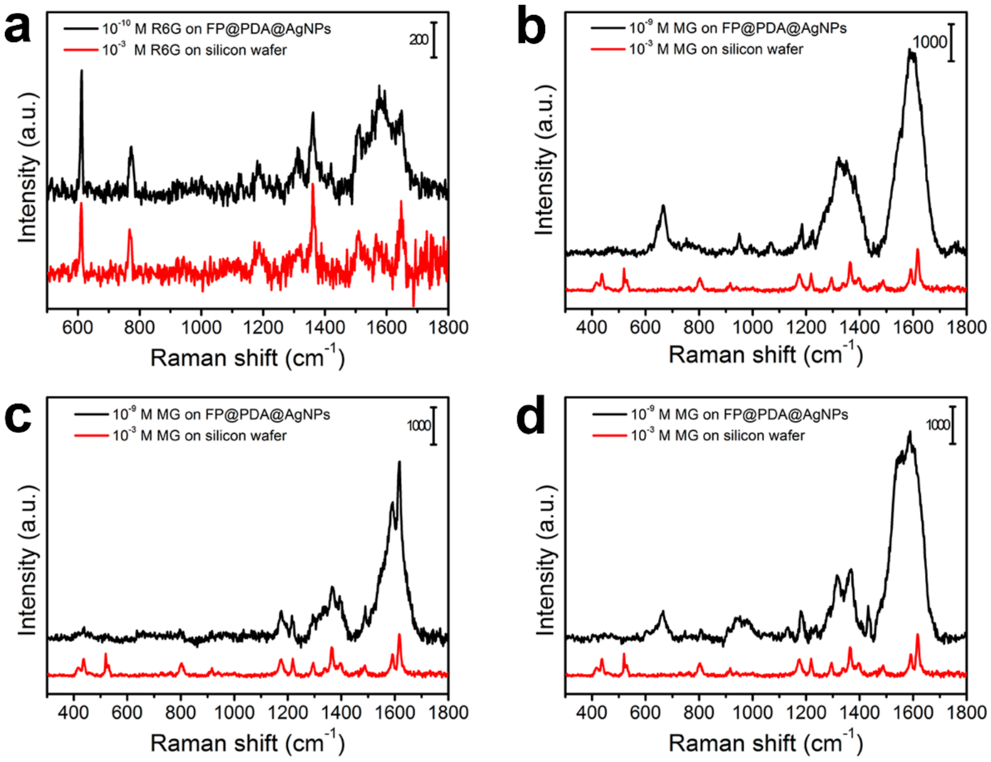

Figure S3. (**a**) SERS spectra (black) for 200 μL 10^{-10} M R6G on 50 mm^2 of FP@PDA@AgNPs and the Raman spectrum (red) for 10^{-3} M R6G on a 50 mm^2 silicon wafer. SERS spectra (black) for 50, 75 and 100 μL of 10^{-9} M MG on 50 mm^2 FP@PDA@AgNPs swabs collected from (**b**) fish scales, (**c**) crab shells and (**d**) shrimp skins, and the Raman spectrum (red) for 50 μL of 10^{-3} M MG on a 50 mm^2 silicon wafer.

Calculation of the Average Enhancement Factor (EF)

The average SERS enhancement factor (EF) of the substrate was calculated from the perspective of the SERS substrate, where the detailed distribution of the singular molecule EF was not considered. Assuming that all of the probe molecules in the laser spot are illuminated and contribute to the SERS and Raman spectra, the EF can be calculated by the following formula:

$$EF = \frac{I_{SERS} N_{RS}}{I_{RS} N_{SERS}} \quad (1)$$

where I_{SERS} and I_{RS} represent the intensities of the same band of the SERS spectra and the Raman spectra (non-SERS), respectively, and N_{SERS} and N_{RS} represent the numbers of molecules on the substrates within the laser spot. In the experiments, a certain volume V_{SERS} of R6G aqueous solution was dispersed over an area of S_{SERS} at a concentration of C_{RS} of C_{SERS} on the FP@PDA@AgNPs substrates. For normal Raman experiments, a certain volume V_{RS} of R6G aqueous solution was dispersed over an area of S_{RS} at a concentration of C_{RS} on a clean silicon wafer. Both of the substrates were dried in air. Therefore, the above equation becomes

$$EF = \frac{I_{SERS}}{I_{RS}} \cdot \frac{S_{SERS} V_{RS} C_{RS}}{S_{RS} V_{SERS} C_{SERS}} \quad (2)$$

Raman measurements were performed under the same experimental conditions（with respect to laser power, laser wavelength, microscope objective/lenses, accumulation time, etc.）

In the experiments, 200 μL of 10^{-10} M R6G aqueous solution was dispersed over an area of 50 mm^2 for the FP@PDA@AgNPs substrates and 200 μL of 10^{-3} M R6G aqueous solution was dispersed over an area of 50 mm^2 for the silicon wafer substrate. Figure S2a shows the SERS spectrum and Raman spectrum for R6G from the above-mentioned substrates. The intensities for the C–C–C in-plane bend mode at 612 cm^{-1} are 401 units for R6G on silicon wafer and 715 units for FP@PDA@AgNPs. The EF of the FP@PDA@AgNPs substrate is calculated as 1.78×10^7.

Figure S2b–d shows the SERS spectra and Raman spectra for MG from the as-prepared substrates. Similarly, the EFs for the FP@PDA@AgNPs on the fish scales, crab shells and shrimp skins surfaces were calculated to be 4.06×10^6, 2.85×10^6 and 1.99×10^6, respectively.
